# Therapeutic potential of taurine in a pigmented rat model of age-related macular degeneration

**DOI:** 10.3389/fopht.2025.1701761

**Published:** 2025-12-01

**Authors:** Mohammed Attia, Tonja Pavlovic, Ashley Muliawan, Farjana Tuya, Maria Mihalatos, Marcin Iwanicki, Jennifer Kang-Mieler

**Affiliations:** 1 Department of Biomedical Engineering, Stevens Institute of Technology, Hoboken, NJ, United States; 2 Department of Chemistry and Chemical Biology, Stevens Institute of Technology, Hoboken, NJ, United States

**Keywords:** dry age-related macular degeneration (AMD), sodium iodate, taurine, oxidative stress, retinal pigment epithelium (RPE), retinal thickness

## Abstract

**Purpose:**

To investigate the potential protective effects of taurine supplementation against retinal degeneration in an animal model of mild dry age-related macular degeneration (AMD).

**Methods:**

To test the effects of a taurine supplement in mild dry AMD, sodium iodate (NaIO_3_)-induced retinal degeneration model was used. Two administration methods, intraperitoneal (IP) and intravenous (IV), were used to deliver NaIO_3_ in pigmented Long Evans rats to generate mild and severe dry AMD, respectively. Structural abnormalities were evaluated *in vivo* using near-infrared (IR) reflectance fundus imaging and optical coherence tomography (OCT). Using the slow progressive mild AMD model, we investigated the neuroprotective effects of oral taurine supplementation (1.5% w/v in drinking water) against NaIO_3_-induced retinal degeneration over 20 weeks. In addition, a human Retinal Pigment Epithelium (RPE, hTERT-RPE1) cell culture model was used to directly assess taurine’s ability to protect against NaIO_3_-related oxidative stress.

**Results:**

The high-dose IV model (80 mg/kg) exhibited extensive and severe retinal damage, with ONL thinning by 64.2% and total retinal thickness (TRT) by 47.6%, predominantly in the peripapillary region. In contrast, the lower-dose IP model (50 mg/kg) displayed milder, more gradual deterioration (outer nuclear layer (ONL) thinning by 19.4% and TRT by 11.5%). Oral taurine supplementation significantly preserved ONL and TRT *in vivo* and supported RPE-1 cell survival, proliferation, and motility, under NaIO_3_ conditions.

**Conclusion:**

Taurine supplementation provided significant structural protection against NaIO_3_-induced damage both *in vivo* and in cell culture, demonstrating its potential as a therapeutic candidate for mitigating mild dry AMD progression.

## 1 Introduction

Age-related macular degeneration (AMD) is one of the leading causes of irreversible vision loss in the aging population worldwide. As of 2020, it was estimated to affect nearly 200 million individuals, with prevalence projected to rise to approximately 288 million by 2040 (1, 2). AMD is categorized into two forms: wet and dry AMD. The wet form, also called neovascular form, is characterized by choroidal neovascularization. The non-neovascular dry form is marked by progressive degeneration of the retinal pigment epithelium (RPE) and photoreceptors (3, 4). The dry form is significantly more prevalent, accounting for approximately 85 – 90% of all AMD cases (5). Despite its high prevalence, therapeutic options for dry AMD remain limited. The Age-Related Eye Disease Studies (AREDS and AREDS2) have demonstrated that dietary supplementation with specific antioxidants such as vitamins C and E, zinc, lutein, and zeaxanthin can delay progression from intermediate to advanced stages of AMD (6). Recently, intravitreal therapies targeting the complement cascade, such as pegcetacoplan (a C3 inhibitor) and avacincaptad pegol (a C5 inhibitor), have been approved by the U.S. Food and Drug Administration (FDA) for the treatment of geographic atrophy (GA), the late stage of dry AMD (7, 8). While these agents slow lesion growth by up to 20% in a dose-dependent manner, they do not restore visual function and require frequent administration (9). To date, there is no effective treatment available to prevent or reverse early-stage dry AMD. Pathologically, early and intermediate stages are characterized by RPE dysfunction, often revealing as drusen accumulation and pigmentary changes. In advanced stages such as GA, there is widespread RPE and photoreceptor loss accompanied by choriocapillaris atrophy (4). The RPE, a monolayer of specialized epithelial cells, is essential for maintaining photoreceptor integrity. It performs critical roles, including the phagocytosis of photoreceptor outer segments, nutrient transport, and participation in the visual cycle (10). Its degeneration disrupts retinal homeostasis and initiates secondary photoreceptor loss, ultimately leading to central vision impairment (11). Oxidative stress is recognized as a major contributor to RPE damage (12). The macula is particularly susceptible due to high oxygen consumption, continuous light exposure, and the abundance of polyunsaturated fatty acids (2, 13). These conditions, combined with aging and environmental insults, generate cumulative oxidative stress that drives RPE dysfunction and degeneration (2). Preclinical studies have demonstrated that oxidative damage reproduces key pathological features associated with dry AMD (4).

Given the central role of oxidative stress in AMD pathogenesis, antioxidant-based interventions have gained attention as potential therapeutic strategies. Taurine, a sulfur-containing β-amino acid, is the most abundant free amino acid in the retina and plays a critical role in maintaining normal retinal structure and function (14). It contributes to cellular osmoregulation, calcium homeostasis, membrane stabilization, and, importantly, oxidative stress defense (15–17). Taurine deficiency is known to cause photoreceptor degeneration and impaired visual function, as demonstrated in early studies involving taurine-deficient cats and rodents (18, 19). Recent studies demonstrate the neuroprotective potential of taurine in various models of retinal degeneration. Systemic taurine supplementation has been shown to reduce oxidative damage and inflammation, preserve photoreceptor survival, and improve visual responses in both chemically and genetically induced models of retinal degeneration (14, 20, 21). In a mouse model of N-methyl-N-nitrosourea (MNU)–induced retinopathy, daily administration of taurine (200 mg/kg) intravenously for 7 consecutive days before and after MNU delivery preserved cone photoreceptor populations, reduced apoptosis and oxidative stress, and improved visual function (20). Similarly, in dystrophic Royal College of Surgeons (RCS) rats with impaired RPE phagocytosis due to a MERTK mutation, taurine provided in drinking water (0.2 M) from postnatal day 21 to 45 resulted in greater photoreceptor survival, enhanced rod and cone responses, reduced microglial infiltration and glial reactivity, and improved phagocytic activity by the retinal pigment epithelium (22). These collective findings indicate that taurine may provide a promising neuroprotective strategy to slow or mitigate the progression of retinal degeneration. Additionally, in humans, oral taurine supplementation (100 mg/kg/day for two years) restored normal plasma taurine levels and stopped progression of retinal degeneration while improving vision in a child with SLC6A6-related inherited retinopathy, demonstrating its potential as a therapeutic strategy for similar disorders (23).

There is currently no animal model that fully recapitulates the progressive features of human dry AMD. Genetic models, such as RCS rats, primarily reflect monogenic causes of degeneration, whereas AMD arises from multifactorial genetic and environmental influences (24, 25). Among experimental systems, sodium iodate (NaIO_3_)–induced models are widely used due to their accessibility, reproducibility, and ability to selectively target the retinal pigment epithelium (RPE). NaIO_3_ promotes intracellular reactive oxygen species (ROS) formation, leading to mitochondrial dysfunction, oxidative stress amplification, and RPE cell death through apoptosis, necroptosis, or necrosis in a dose-dependent manner (26–30). Additional toxicity arises from interactions with melanin, generating glyoxylate and inhibiting key metabolic enzymes (30). Loss of RPE integrity subsequently compromises photoreceptor survival, manifesting as thinning of the outer nuclear layer, while associated immune activation, including complement pathways and microglial infiltration, further propagates retinal inflammation and degeneration (26, 31).

Previous studies have demonstrated that NaIO_3_ injection in rodents induces a spectrum of retinal damage, ranging from localized RPE disruption to widespread geographic atrophy-like lesions (4). However, the effects of NaIO_3_ vary across studies, influenced by factors such as the administered dose, route of delivery, animal strain, and age. These factors significantly influence the severity and distribution of retinal lesions. In C57BL/6J mice, intravenous administration of NaIO_3_ resulted in dose-dependent structural alterations: 10–20 mg/kg caused no detectable long-term retinal thinning by OCT, 30 mg/kg induced moderate structural degeneration with visible thinning of the RPE and outer retina, and doses ≥ 40 mg/kg produced extensive retinal atrophy characterized by pronounced disruption and thinning across multiple retinal layers (4). A previous study in Sprague-Dawley rats demonstrated intravenous delivery of 40 mg/kg NaIO_3_ produced degenerative lesions in the outer retina, while higher doses resulted in more acute damage (32).

This study investigated the potential of taurine to protect against retinal degeneration associated with oxidative stress. *In vitro*, we examined whether taurine could preserve the viability, proliferative capacity, and regenerative potential of cultured human RPE cells following NaIO_3_-induced oxidative stress. *In vivo*, we assessed whether prolonged oral taurine supplementation could mitigate structural retinal damage in a pigmented rat model of mild dry AMD.

## 2 Materials and methods

### 2.1 Animal preparation

All animal procedures were conducted in accordance with protocols approved by the Institutional Animal Care and Use Committee (IACUC) at Stevens Institute of Technology (Protocol #2022-002). Experimental protocols adhered to the ARVO Statement for the Use of Animals in Ophthalmic and Vision Research. Male Long-Evans rats (225–275 g; Charles River Laboratories, Fairfield, NJ, USA) were housed under standard laboratory conditions with a 12-hour light/dark cycle and given ad libitum access to food and water. Prior to all procedures and imaging sessions, rats were anesthetized via intraperitoneal injection of ketamine hydrochloride (80 mg/kg; Dechra Veterinary Products, Overland Park, KS, USA) and xylazine (10 mg/kg; Cronus Pharma LLC, East Brunswick, NJ, USA). Topical anesthesia of the cornea was achieved using proparacaine hydrochloride ophthalmic drops (Alcon Laboratories Inc., Fort Worth, TX, USA). Pupillary dilation was induced using topical phenylephrine and atropine eye drops (both from Lifestar Pharma LLC, Mahwah, NJ, USA). To maintain physiological conditions during the experiments, animals were placed on a heated stage to sustain core body temperature at approximately 37°C.

### 2.2 Experimental design and sodium iodate-induced retinal degeneration

Sodium iodate (Thermo Scientific Chemicals, Waltham, MA, USA) was freshly prepared at a concentration of 1% (w/v) in sterile saline immediately before administration. To evaluate the impact of NaIO_3_ dose and route of administration on retinal degeneration severity and progression, six rats were randomly assigned to the following groups: (1) Healthy Control Group, sterile saline treatment administered intraperitoneally; (2) Gradual mild AMD Group, received intraperitoneal (IP) injection of 50 mg/kg NaIO_3_; and (3) Rapid severe AMD Group, received intravenous (IV) injection of 80 mg/kg NaIO_3_ via the lateral tail vein. The mild AMD model, induced via IP injection of NaIO_3_, was subsequently used to assess the therapeutic efficacy of oral taurine supplementation.

### 2.3 Assessment of taurine protection effect

#### 2.3.1 Cell culture and taurine treatments

Human retinal pigment epithelial cells (hTERT-RPE1; ATCC, Manassas, VA, USA) were cultured in a 1:1 mixture of Dulbecco’s Modified Eagle Medium and Ham’s F-12 (DMEM/F-12), supplemented with 5% fetal bovine serum and 1% penicillin–streptomycin. Cultures were maintained at 37°C in a humidified incubator with 5% CO_2_. For experiments, cells were seeded and grown to approximately 50–70% confluence before treatment. Taurine (2 mM; Thermo Scientific Chemicals) was added to the culture medium 24 hours prior to sodium iodate exposure to allow cellular uptake. Cells were divided into three groups: an untreated control group, a group exposed to 2 mM NaIO_3_, and a group co-treated with 2 mM NaIO_3_ and 2 mM taurine. The selection of 2 mM NaIO_3_ was to induce a sublethal level of oxidative stress in human RPE cell lines that is optimal for evaluating cytoprotective interventions (33).

#### 2.3.2 Cell proliferation assay

To monitor cell growth over time, cells were seeded at equal densities and allowed to adhere overnight. Cell counts were recorded over a 7-day period using an automated cell counter (Luna II, Logos Biosystems, Annandale, VA, USA). This assay was used to assess the impact of NaIO_3_ and taurine on RPE cell proliferation.

#### 2.3.3 Propidium iodide staining and flow cytometry

Cell viability and death were further evaluated using PI staining. Treated cells were harvested, washed with PBS containing 2% bovine serum albumin (BSA), and incubated with 5 μg/mL propidium iodide for 30 minutes at room temperature. Samples were analyzed using an Attune NxT flow cytometer (Thermo Fisher Scientific). Viable cells excluded PI and were considered PI-negative, whereas non-viable cells took up the dye and were recorded as PI-positive.

#### 2.3.4 Wound healing assay

To assess regenerative capacity following injury, RPE cells were cultured to full confluence and treated with taurine, with or without NaIO_3_. A linear scratch was introduced using a sterile 10 μL pipette tip. Following PBS washing to remove detached cells, treatment-containing media were reapplied. Wound closure was monitored for 24 hours using time-lapse live-cell microscopy (BioTek Lionheart FX, Winooski, VT, USA). Images were analyzed using ImageJ software, and wound closure was quantified by comparing the remaining wound area at each time point to the initial wound size (34).

#### 2.3.5 Taurine treatment in animal study

Taurine was administered in drinking water at a concentration of 1.5% (w/v), following previously established dosing protocols (35). Four rats were assigned to the taurine treatment group. Half of these animals began taurine supplementation two weeks prior to NaIO_3_ injection, while the remaining half initiated taurine treatment two weeks after NaIO_3_ administration. As both pre- and post-treatment subgroups demonstrated comparable retinal outcomes, they were combined into a single taurine treatment group for analysis.

### 2.4 Retinal imaging and quantification

Retinal structural assessment was performed using a confocal scanning laser ophthalmoscope (cSLO) equipped with optical coherence tomography (OCT) (SPECTRALIS; Heidelberg Engineering, Heidelberg, Germany). Imaging was conducted biweekly starting at baseline (prior to NaIO_3_ injection) and continued for 6 weeks in the dose- and route-dependent NaIO_3_-induced retinal degeneration study (Section 2.2), and for 20 weeks in the oral taurine treatment efficacy study (Section 2.3.5), following the respective experimental design. Retinal thickness was evaluated on OCT scans using ImageJ software (NIH, Bethesda, MD, USA). For each eye, 14–20 measurements of the outer nuclear layer (ONL) and total retinal thickness (TRT) were obtained from each side of the optic disc. These measurements were averaged per eye, and group means were calculated across all eyes under the same experimental condition.

### 2.5 Statistical analysis

All quantitative data are reported as mean ± standard deviation of the mean (SD). Statistical analysis was conducted using GraphPad Prism software. Differences in cell behavior were assessed using one-way ANOVA that was used for comparison among three groups, followed by the Holm–Sidak *post hoc* test for multiple comparisons. Unpaired *t*-tests were employed for comparisons between the control and test group. For comparisons between the control and individual treatment groups at specific time points, multiple unpaired *t*-tests were conducted. Kruskal–Wallis test followed by Dunn’s test for multiple comparisons was utilized to compare differences in normalized retinal thickness after taurine administration. Statistical significance was set at *P* < 0.05, and significance was indicated in graphical data using asterisks.

## 3 Results

### 3.1 Taurine preserves human RPE cell viability and regeneration under oxidative stress

**Figure 1:**
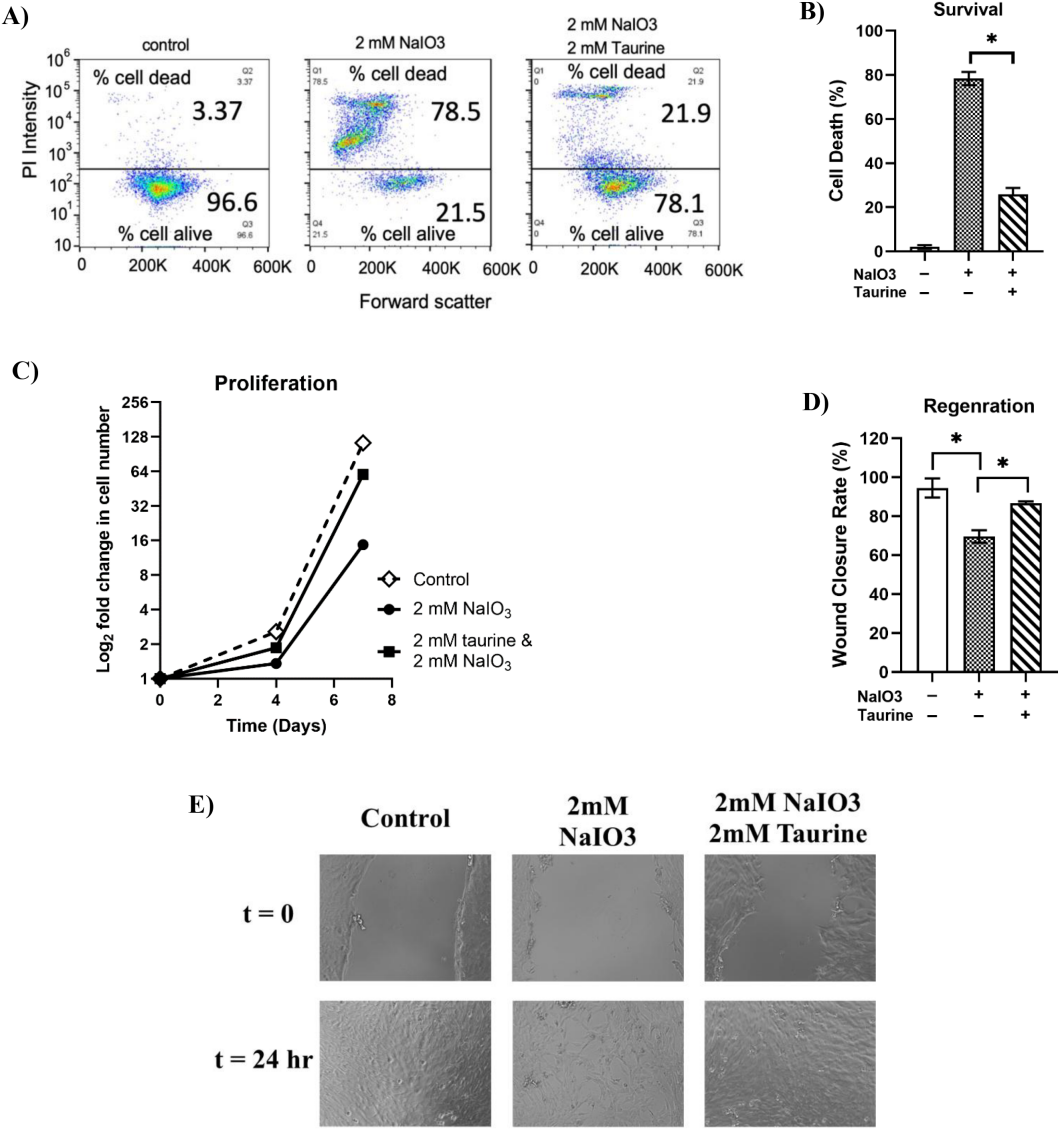
**(A, B)** Flow cytometry analysis of propidium iodide signal inferring cell death, **(C)** Cell proliferation data over seven days, **(D)** Quantification of wound closure percentage 24 hours post-scratch injury, **(E)** Representative images of scratch assays at 0 and 24 hours across treatment groups. Error bars are standard deviation. *statistically significant (p < 0.05).

To evaluate the protective effect of taurine against NaIO_3_-induced toxicity, a series of *in vitro* assays were conducted using cultured human retinal pigment epithelial (hTERT-RPE1 cell line). Flow cytometry analysis of propidium iodide staining demonstrated that exposure to 2 mM NaIO_3_ resulted in approximately 78.5 ± 3.0% cell death, as determined by propidium iodide staining followed by flow cytometry analysis (**Figures 1A, B**). Co-treatment with 2 mM taurine markedly reduced cell death to 21.9 ± 2.8%, indicating a substantial cytoprotective effect.

Exposure of RPE cells to 2 mM sodium iodate markedly reduced their proliferation capacity, indicating that NaIO_3_ inhibits RPE cell division (**Figure 1C**). However, co-treatment with 2 mM taurine effectively rescued this inhibitory effect, restoring cell growth to levels comparable to untreated controls. This suggests that taurine mitigates NaIO_3_-induced suppression of cell proliferation. In wound healing assays, NaIO_3_ significantly impaired the regenerative ability of the RPE monolayer, as shown by delayed wound closure following mechanical injury. In contrast, taurine treatment markedly improved the rate of wound closure after NaIO_3_ insult from 69.6 ± 3.2% to 86.6 ± 1.1% and restored regenerative capacity (**Figures 1D, E**), indicating preservation of epithelial repair mechanisms. These functional assessments demonstrate that exposure of RPE cells to NaIO_3_ disrupts survival, proliferation and regeneration of RPE cells and highlight taurine’s cytoprotective effects to support RPE cell viability under oxidative stress.

### 3.2 Comparative retinal degeneration in mild and severe dry AMD models

**Figure 2:**
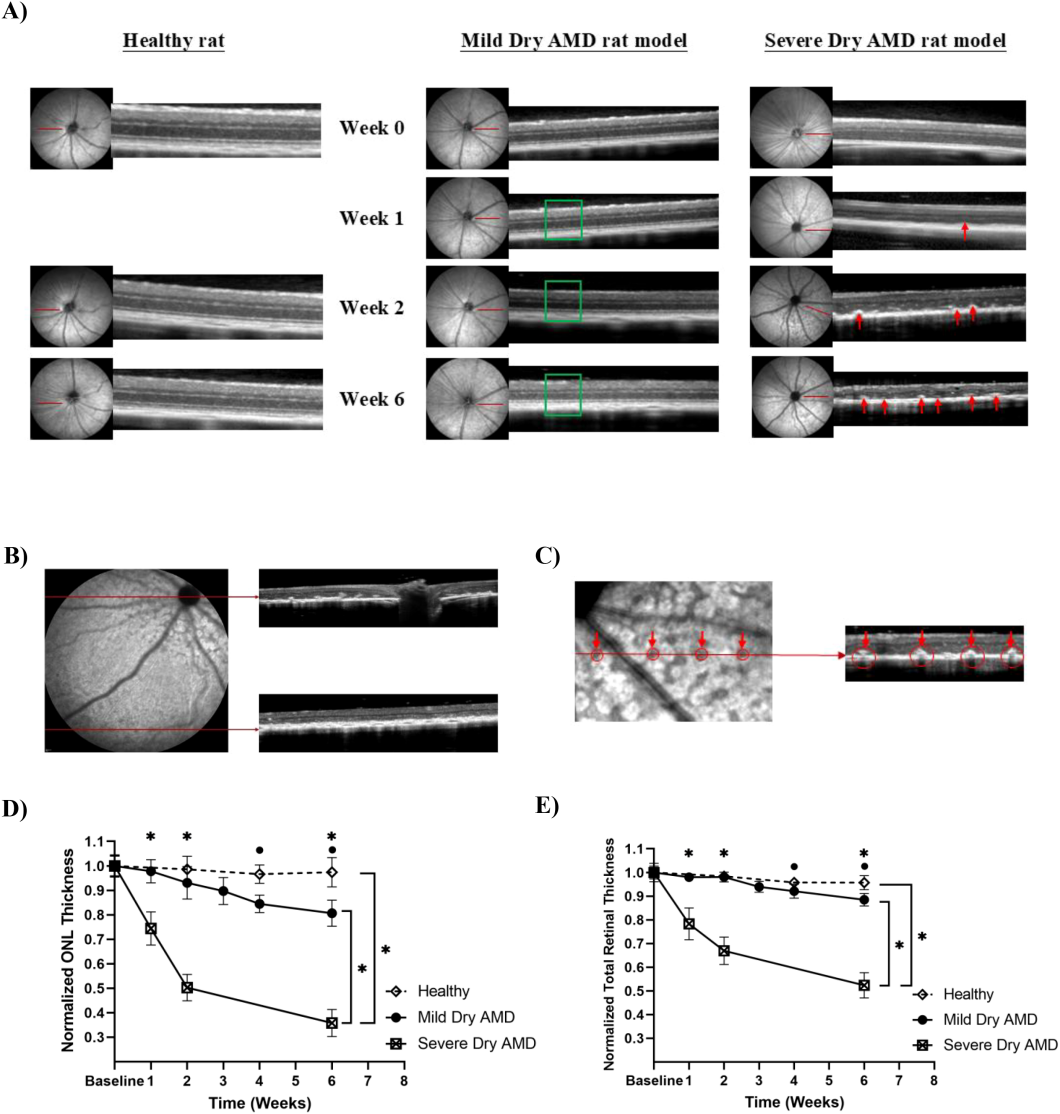
**(A)** The IR reflectance for the sodium iodate-induced mild and severe dry AMD rat model, and the cross-sectional OCT images showing the appearance of retinal layers from a representative experimental animal. The green box highlights the preserved retinal structure without hyperreflective abnormalities, whereas the red arrows indicate dome-shaped hyperreflective foci between the ONL and photoreceptor segments, representing early degenerative changes observed at week 1 and progressing over time, **(B)** Spatial pattern of retinal degeneration highlighting regional variability in susceptibility to oxidative damage, with the peripapillary zone showing greater degeneration compared to the peripheral retina, **(C)** Dark blots in IR reflectance images are correlated to the degenerative profiles in OCT images, **(D, E)** Normalized ONL and total retinal thickness measurements for mild and severe dry AMD rat model. Error bars are standard deviation. *statistically significant (p < 0.05).

To investigate how sodium iodate (NaIO_3_) dose and administration route influence the onset and severity of retinal degeneration, two models of dry age-related macular degeneration (AMD) were developed in Long-Evans rats. A lower-dose intraperitoneal (IP) model using 50 mg/kg NaIO_3_ (low-dose IP) was used to mimic slow progressive mild degeneration, while a high-dose intravenous (IV) model using 80 mg/kg NaIO_3_ (high-dose IV) was designed to replicate advanced dry AMD. Infrared (IR) reflectance fundus imaging captured pronounced differences in disease progression between the two models. Dark patches in the IR images represent localized RPE atrophy and melanin loss (36). Rats in the low-dose IP group did not exhibit such dark regions throughout the observation period (**Figure 2A**). In contrast, in the high-dose IV group, extensive dark patches were evident as early as one week after NaIO_3_ injection (**Figure 2A**). These dark patches were localized predominantly around the optic nerve head and gradually became less prominent toward the periphery, as shown in the IR image in **Figure 2B**. OCT imaging revealed additional structural differences. While rats in the low-dose IP group exhibited normal retinal structure without hyperreflective abnormalities (green box), those in the high-dose IV group displayed dome-shaped hyperreflective foci between the outer nuclear layer (ONL) and the photoreceptor segments as early as week 1 (red arrows) (**Figure 2A**). They result from increased scattering by cellular retinal structural disorganization at this region and intensified over time (37). Notably, the distribution of these hyperreflective lesions mirrored the regional pattern of IR abnormalities, being most prominent around the optic nerve head and less evident toward the periphery (**Figure 2B**). These lesions were associated with thinning of the ONL, disorganization of the photoreceptor outer segments, and progressive loss of total retinal thickness (32). Quantitative analysis showed that the rats in the low-dose IP group exhibited a more moderate reduction in ONL thickness of 19.4 ± 5.3% compared to 64.2 ± 5.5% in the high-dose IV group by week six (**Figure 2D**). Similarly, total retinal thickness (TRT) decreased by 11.5 ± 2.6% in the low-dose IP group, while the high-dose IV group showed a severe decline of 47.6 ± 5.4% (**Figure 2E**). No considerable changes were observed in the ONL (< 2.6%) or TRT (< 4.3%) of the healthy control group throughout the experiment. **Figure 2C** further illustrates the spatial correspondence between planar dark areas on IR reflectance imaging and focal hyperreflective lesions in OCT cross-sections. Red arrows in the IR images indicate localized signal loss along the scan line, while matching arrows in the OCT images highlight hyperreflective regions that align anatomically with the IR findings. This colocalization supports a consistent association between reflectance abnormalities and structural changes in the retina, particularly involving RPE atrophy and photoreceptor layer disorganization. Based on the current findings, the low-dose IP group was used to test the oral taurine neuroprotective effects.

### 3.3 Oral taurine supplementation preserves retinal structure in a rat model of mild dry AMD

**Figure 3:**
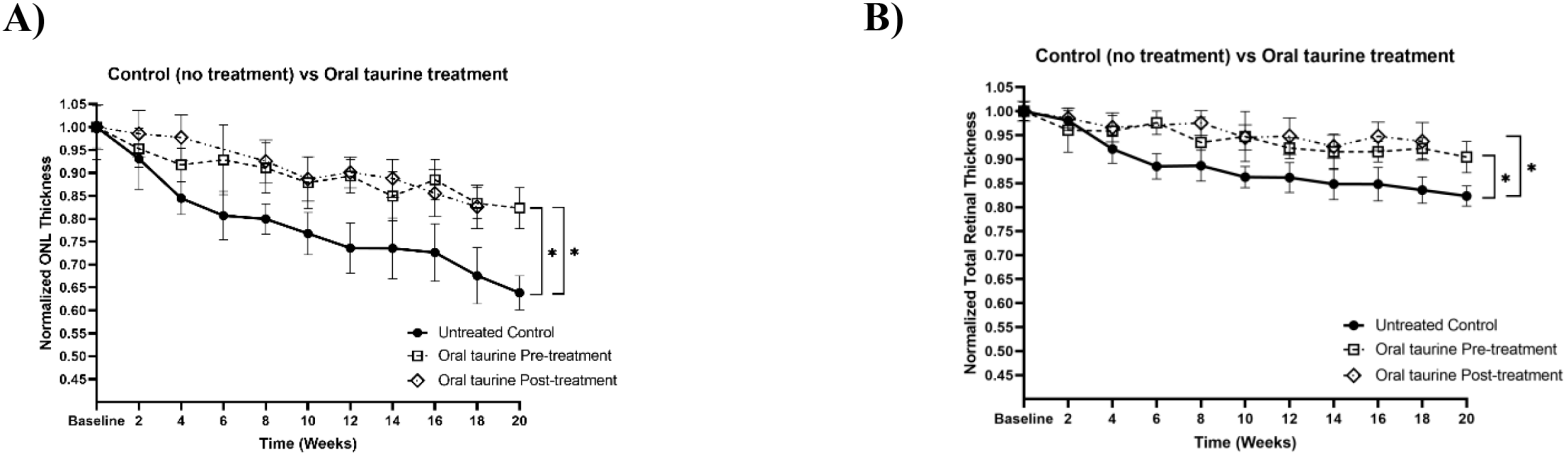
**(A, B)** Normalized ONL and total retinal thickness measurements for the untreated control group (n = 3 rats) versus oral taurine pre- (n = 2 rats) and post-treatment (n = 2 rats) groups. Statistical differences were determined using Kruskal-Wallis test followed by Dunn’s test for multiple comparisons (*p* < 0.05). Error bars are standard deviation. *statistically significant (p < 0.05). *statistically significant (p < 0.05).

*In vivo* experiments were carried out to assess the structural preservation of the retina following oral taurine supplementation in rats exposed to 50 mg/kg intraperitoneal NaIO_3_. Two taurine administration schedules were evaluated: one beginning two weeks prior to NaIO_3_ injection (pre-treatment) and one beginning two weeks after injection (post-treatment). No significant difference was observed between these two regimens in terms of TRT (p = 0.15) and ONL (p = 0.55) preservation (**Figures 3A, B**). Longitudinal retinal measurements showed that both pre- and post- oral taurine significantly mitigated ONL thinning compared to the untreated control group. Specifically, ONL thickness reduction was 17.7 ± 4.5% in the pre-treated group and 17.4 ± 4.8% in the post-treated group, compared to 36.1 ± 3.7% in the untreated degeneration group (**Figure 3A**). TRT was similarly preserved, exhibiting reductions of 9.6 ± 3.3% (pre-treated) and 6.3 ± 4.0% (post-treated) whereas the untreated group demonstrated a 17.7 ± 2.1% reduction (**Figure 3B**). These data indicate that oral taurine mitigated NaIO_3_-induced retinal thinning, mainly ONL layer, and preserved structural integrity of the retinal layers under oxidative stress conditions.

## 4 Discussion

This study evaluated the neuroprotective potential of oral taurine supplementation, a compound known for its antioxidant and membrane-stabilizing properties. By assessing the extent of retinal preservation in taurine-treated animals under mild conditions modeling slow progressive dry age-related macular degeneration (AMD), this work provides insights into its therapeutic utility under oxidative stress conditions for slowing or mitigating dry AMD progression. To establish an appropriate model for assessing taurine’s therapeutic effects, we investigated the retinal effects of sodium iodate (NaIO_3_) administration in a pigmented rodent model to establish and differentiate between mild and severe phenotypes of dry AMD. By employing two distinct dosing regimens, we aimed to control the progression of RPE and photoreceptor degeneration.

To explore taurine’s direct cytoprotective effects *in vitro*, we first examined its impact on human RPE cells exposed to NaIO_3_ (**Figure 1**). Taurine treatment maintained cellular viability under NaIO_3_-induced cytotoxic stress, suggesting a substantial capacity to enhance RPE resilience. While oxidative stress was not directly assessed in this study, the observed protective effects align with NaIO_3_’s known mechanism of toxicity, which primarily involves oxidative and mitochondrial damage to RPE cells. Taurine’s cytoprotective actions have been extensively documented and are attributed to several complementary pathways, including modulation of intracellular calcium homeostasis, maintenance of mitochondrial membrane potential, and suppression of caspase-dependent apoptosis (38, 39). Importantly, taurine scavenges hypochlorous acid, a potent oxidant produced by neutrophils, forming taurine chloramine, a less reactive compound that exerts both antioxidative and anti-inflammatory effects (40, 41). Additionally, taurine mitigates mitochondrial superoxide generation, thereby reducing one of the major intracellular sources of reactive oxygen species (42).

Moreover, our results show the preservation of cell proliferation and wound healing capacity in taurine-treated RPE cultures, which suggests that taurine not only prevents cytotoxicity but also actively supports cellular regenerative functions. Taurine-treated cells maintained growth rates comparable to untreated controls, indicating sustained mitotic activity even under insult. In addition, taurine significantly improved wound closure in scratch assays, reflecting enhanced cell migration and monolayer repair. Previous reports showed that taurine promotes proliferation in human and rabbit RPE cells (43), supporting its role in cellular renewal. Therefore, the improved cell viability and migration observed in our study following taurine treatment are consistent with a generalized cytoprotective response, involving but not limited to antioxidant mechanisms.

Building on these *in vitro* findings, we next established and compared two distinct NaIO_3_-induced retinal degeneration models in pigmented rats to evaluate how the severity of retinal degeneration influences the appearance of dry AMD-like pathology and to identify an appropriate model for assessing taurine’s *in vivo* protective potential. Only male rats were used to provide a consistent baseline for evaluation by avoiding variability related to hormonal cycles and estrogen-associated antioxidant effects (44, 45). Future studies should investigate sex as a biological variable, since previous reports showed that sex-specific differences in NaIO_3_ susceptibility (46, 47).

The method of NaIO_3_ delivery has a critical influence on the extent and rate of retinal injury. Consistent with earlier findings, intraperitoneal (IP) injection results in slower systemic absorption and milder toxicity, whereas intravenous (IV) administration produces a rapid increase in plasma concentration and more severe, widespread damage (32). Therefore, we used 50 mg/kg IP NaIO_3_ to model gradual mild retinal degeneration and 80 mg/kg IV NaIO_3_ to mimic more rapid severe disease. Evidence of RPE atrophy was supported by persistent dark regions on near- IR fundus imaging, resulting from melanin depletion that contributes to IR reflectivity (**Figure 2A**) (36). OCT imaging further revealed early signs of outer retinal disruption, including hyperreflective foci and disorganization in the outer nuclear layer and photoreceptor segments, consistent with localized cell damage. NaIO_3_ mainly exerts selective toxicity toward RPE, leading to RPE cell loss and atrophy (48). As a result, it can no longer perform its normal functions in maintaining photoreceptor health through phagocytosis of photoreceptor outer segments, nutrient transport, and oxidative stress regulation. This leads to secondary damage in the photoreceptors through disorganization of the photoreceptor layer and progressive thinning of the ONL, which contains the photoreceptor nuclei. As the injury progressed, these alterations evolved into more pronounced structural changes, including retinal thinning and disruption of the photoreceptor integrity, aligning with the degenerative trajectory observed in advanced dry AMD. This interpretation is consistent with established mechanisms described in previous studies, providing contextual support for the observed findings.

The spatial pattern of retinal degeneration, seen as dark patches in IR images and hyperreflective lesions in OCT scans, reflects regional differences in sensitivity to oxidative stress. Greater damage was observed in the peripapillary region compared to the peripheral retina, suggesting this central zone is more vulnerable to NaIO_3_-induced injury (**Figure 2B**). This pattern corresponds to higher metabolic and oxygen demand near the optic nerve head, prompting central retinal regions to greater ROS accumulation as reported in previous study (49). Regional variations in antioxidant enzyme activity may also contribute, as human retinal studies have shown lower catalase and glutathione peroxidase activity in the macula compared to peripheral regions, suggesting that zones with lower antioxidant capacity are less prepared to neutralize ROS and hence are more susceptible to oxidative insult (50).

A clear correspondence between the planar dark areas observed in IR imaging and dome-shaped hyperreflective lesions in OCT (**Figure 2C**) highlights the link between imaging abnormalities and underlying structural damage within the retina. Regions of reduced IR reflectivity are commonly associated with RPE atrophy and melanin loss. These areas align closely with localized disruptions in the photoreceptor layer, suggesting that damage to the RPE compromises the health and stability of the overlying photoreceptors. In contrast, the low-dose IP model exhibited preserved retinal architecture and normal IR reflectivity, highlighting the critical role of dose and administration route in determining disease severity and progression.

Longitudinal OCT analysis demonstrated distinct thinning trajectories between the IV and IP NaIO_3_ models (**Figure 2D**). The high-dose IV model showed rapid ONL and total retinal thickness (TRT) loss reflecting acute RPE destruction and widespread photoreceptor death. The gradual thinning observed in the low-dose IP model reflected a more chronic process, representative of slow progressive mild AMD (26). ONL thinning appeared earlier and progressed faster than overall TRT reduction, supporting its role as an early biomarker of photoreceptor degeneration. The more pronounced TRT decline in the IV model suggests damage extended into inner retinal layers, likely due to extensive RPE loss and secondary degeneration across multiple layers, driven by severe NaIO_3_-induced oxidative stress (4). Conversely, the slower rate of TRT decline in the lower-dose IP group suggests that inner retinal layers were less affected, as lower NaIO_3_ doses primarily affect the RPE and outer retina, while inner retinal layers demonstrate greater preservation due to being both functionally less dependent on RPE support and anatomically more distant from the site of toxicity (4). Previous studies confirm that extensive NaIO_3_ injury affects not only photoreceptors but also inner retinal neurons, including dopaminergic amacrine and retinal ganglion cells, through caspase and calpain activation (28). The structural deterioration in our IV model is consistent with these reports, demonstrating system-wide degeneration, whereas the IP model better represents a manageable oxidative stress level suitable for therapeutic testing.

The route-dependent variation in damage severity can be explained by pharmacokinetics. Intravenous administration produces a sharp plasma spike and immediate retinal exposure, resulting in rapid oxidative insult, while intraperitoneal delivery produces lower peak concentrations and slower systemic distribution (32, 51). Moreover, IV NaIO_3_ doses above 50 mg/kg have been linked to hepatic and renal injury (32). This highlights the importance of dose selection and route of administration for minimizing systemic effects and improving the ocular outcome assessments.

Following establishment of the slow progressive IP model, taurine’s protective efficacy was evaluated under these milder retinal degeneration conditions. *In vivo*, oral taurine supplementation mitigated ONL and TRT thinning in rats subjected to NaIO_3_-induced stress. Although specific oxidative markers were not quantified, the preserved retinal architecture is consistent with taurine’s antioxidative, anti-inflammatory, and membrane-stabilizing properties. The outcome agrees with previous reports showing that taurine supplementation preserves photoreceptor structure and synaptic organization in other various models of retinal degeneration, including RCS rats and MNU-exposed mice (20, 22). These studies collectively support the view that taurine’s beneficial effects arise through antioxidative, anti-inflammatory, and synapse-preserving mechanisms. Our structural findings are consistent with the broader evidence base supporting the cytoprotective role of taurine.

In conclusion, this study highlights the therapeutic potential of taurine in protecting against retinal degeneration, particularly under conditions that mimic slow progressive dry AMD. Using a mild NaIO_3_-induced model in pigmented rats, taurine supplementation preserved retinal structure and demonstrated cytoprotective effects consistent with resistance to oxidative damage, thus highlighting taurine as a potential candidate for further investigation in retinal degeneration therapy. While the current findings primarily reflect anatomical preservation, future studies incorporating functional assessments and long-term treatment paradigms will be essential to fully explain taurine’s therapeutic potential.

## Data Availability

The original contributions presented in the study are included in the article/supplementary material. Further inquiries can be directed to the corresponding author.

## References

[1] Wong WL Su X Li X Cheung CMG Klein R Cheng C-Y . Global prevalence of age-related macular degeneration and disease burden projection for 2020 and 2040: a systematic review and meta-analysis. Lancet Global Health. (2014) 2:e106–16. doi: 10.1016/S2214-109X(13)70145-1, PMID: 25104651

[2] Maurya M Bora K Blomfield AK Pavlovich MC Huang S Liu C-H . Oxidative stress in retinal pigment epithelium degeneration: From pathogenesis to therapeutic targets in dry age-related macular degeneration. Neural Regenerat Res. (2023) 18:2173–81. doi: 10.4103/1673-5374.369098, PMID: 37056126 PMC10328284

[3] Bandello F Sacconi R Querques L Corbelli E Cicinelli MV Querques G . Recent advances in the management of dry age-related macular degeneration: a review. F1000Research. (2017) 6:245. doi: 10.12688/f1000research.10664.1, PMID: 28529701 PMC5428517

[4] Koster C van den Hurk KT Ten Brink JB Lewallen CF Stanzel BV Bharti K . Sodium-iodate injection can replicate retinal degenerative disease stages in pigmented mice and rats: Non-invasive follow-up using OCT and ERG. Int J Mol Sci. (2022) 23:2918. doi: 10.3390/ijms23062918, PMID: 35328338 PMC8953416

[5] Schultz NM Bhardwaj S Barclay C Gaspar L Schwartz J . Global burden of dry age-related macular degeneration: a targeted literature review. Clin Ther. (2021) 43:1792–818. doi: 10.1016/j.clinthera.2021.08.011, PMID: 34548176

[6] Gorusupudi A Nelson K Bernstein PS . The age-related eye disease 2 study: micronutrients in the treatment of macular degeneration. Adv Nutr. (2017) 8:40–53. doi: 10.3945/an.116.013177, PMID: 28096126 PMC5227975

[7] Liao DS Grossi FV El Mehdi D Gerber MR Brown DM Heier JS . Complement C3 inhibitor pegcetacoplan for geographic atrophy secondary to age-related macular degeneration: a randomized phase 2 trial. Ophthalmology. (2020) 127:186–95. doi: 10.1016/j.ophtha.2019.07.011, PMID: 31474439

[8] Danzig CJ Khanani AM Loewenstein A . C5 inhibitor avacincaptad pegol treatment for geographic atrophy: A comprehensive review. Immunotherapy. (2024) 16:779–90. doi: 10.1080/1750743X.2024.2368342, PMID: 39073397 PMC11457614

[9] Cruz-Pimentel M Wu L . Complement inhibitors for advanced dry age-related macular degeneration (geographic atrophy): some light at the end of the tunnel? J Clin Med. (2023) 12:5131. doi: 10.3390/jcm12155131, PMID: 37568533 PMC10420150

[10] Wang S Li W Chen M Cao Y Lu W Li X . The retinal pigment epithelium: functions and roles in ocular diseases. Fundam Res. (2023) 4:1710–18. doi: 10.1016/j.fmre.2023.08.011, PMID: 39734536 PMC11670733

[11] Wang K Liu Y Li S Zhao N Qin F Tao Y . Unveiling the therapeutic potential and mechanisms of stanniocalcin-1 in retinal degeneration. Survey Ophthalmol. (2024) 70:106–20. doi: 10.1016/j.survophthal.2024.08.001, PMID: 39270826

[12] Datta S Cano M Ebrahimi K Wang L Handa JT . The impact of oxidative stress and inflammation on RPE degeneration in non-neovascular AMD. Prog Retinal eye Res. (2017) 60:201–18. doi: 10.1016/j.preteyeres.2017.03.002, PMID: 28336424 PMC5600827

[13] Chaudhuri M Hassan Y Vemana PPSB Pattanashetty MSB Abdin ZU Siddiqui HF . Age-related macular degeneration: an exponentially emerging imminent threat of visual impairment and irreversible blindness. Cureus. (2023) 15:e39624. doi: 10.7759/cureus.39624, PMID: 37388610 PMC10300666

[14] Froger N Moutsimilli L Cadetti L Jammoul F Wang Q-P Fan Y . Taurine: the comeback of a neutraceutical in the prevention of retinal degenerations. Prog Retinal eye Res. (2014) 41:44–63. doi: 10.1016/j.preteyeres.2014.03.001, PMID: 24721186

[15] Pasantes-Morales H Cruz C . Taurine: a physiological stabilizer of photoreceptor membranes. Prog Clin Biol Res. (1985) 179:371–81., PMID: 3903757

[16] El Idrissi A Trenkner E . Growth factors and taurine protect against excitotoxicity by stabilizing calcium homeostasis and energy metabolism. J Neurosci. (1999) 19:9459–68. doi: 10.1523/JNEUROSCI.19-21-09459.1999, PMID: 10531449 PMC6782936

[17] Schaffer S Takahashi K Azuma J . Role of osmoregulation in the actions of taurine. Amino Acids. (2000) 19:527–46. doi: 10.1007/s007260070004, PMID: 11140357

[18] Schmidt SY . (1981). Taurine in retinas of taurine-deficient cats and RCS rats. The Effects of Taurine on Excitable Tissues, in: Proceedings of the 21st Annual AN Richards Symposium of the Physiological Society of Philadelphia, Valley Forge, Pennsylvania, April 23–24, 1979, Berlin, Germany: Springer Nature.

[19] Heller-Stilb B van Roeyen C Rascher K Hartwig H-G Huth A Seeliger MW . Disruption of the taurine transporter gene (taut) leads to retinal degeneration in mice. FASEB J. (2002) 16:1–18. doi: 10.1096/fj.01-0691fje, PMID: 11772953

[20] Tao Y He M Yang Q Ma Z Qu Y Chen W . Systemic taurine treatment provides neuroprotection against retinal photoreceptor degeneration and visual function impairments. Drug Design Dev Ther. (2019) 13:2689. doi: 10.2147/DDDT.S194169, PMID: 31496648 PMC6689665

[21] Lee D Smith LE . Therapeutic effects of taurine and histidine supplementation in retinal diseases. Life. (2024) 14:1566. doi: 10.3390/life14121566, PMID: 39768274 PMC11676320

[22] Martínez-Vacas A Di Pierdomenico J Gallego-Ortega A Valiente-Soriano FJ Vidal-Sanz M Picaud S . Systemic taurine treatment affords functional and morphological neuroprotection of photoreceptors and restores retinal pigment epithelium function in RCS rats. Redox Biol. (2022) 57:102506. doi: 10.1016/j.redox.2022.102506, PMID: 36270186 PMC9583577

[23] Ansar M Ranza E Shetty M Paracha SA Azam M Kern I . Taurine treatment of retinal degeneration and cardiomyopathy in a consanguineous family with SLC6A6 taurine transporter deficiency. Hum Mol Genet. (2020) 29:618–23. doi: 10.1093/hmg/ddz303, PMID: 31903486 PMC7068170

[24] Stradiotto E Allegrini D Fossati G Raimondi R Sorrentino T Tripepi D . Genetic aspects of age-related macular degeneration and their therapeutic potential. Int J Mol Sci. (2022) 23:13280. doi: 10.3390/ijms232113280, PMID: 36362067 PMC9653831

[25] Bhumika NS Bora PS . Genetic insights into age-related macular degeneration. Biomedicines. (1479) 12:1479. doi: 10.3390/biomedicines12071479, PMID: 39062052 PMC11274963

[26] Kannan R Hinton DR . Sodium iodate induced retinal degeneration: new insights from an old model. Neural Regenerat Res. (2014) 9:2044–5. doi: 10.4103/1673-5374.147927, PMID: 25657718 PMC4316465

[27] Zhou P Kannan R Spee C Sreekumar PG Dou G Hinton DR . Protection of retina by αB crystallin in sodium iodate induced retinal degeneration. PloS One. (2014) 9:e98275. doi: 10.1371/journal.pone.0098275, PMID: 24874187 PMC4038555

[28] Balmer J Zulliger R Roberti S Enzmann V . Retinal cell death caused by sodium iodate involves multiple caspase-dependent and caspase-independent cell-death pathways. Int J Mol Sci. (2015) 16:15086–103. doi: 10.3390/ijms160715086, PMID: 26151844 PMC4519888

[29] Hanus J Anderson C Sarraf D Ma J Wang S . Retinal pigment epithelial cell necroptosis in response to sodium iodate. Cell Death Discov. (2016) 2:1–9. doi: 10.1038/cddiscovery.2016.54, PMID: 27551542 PMC4979458

[30] Espitia-Arias MD de la Villa P Paleo-García V Germain F Milla-Navarro S . Oxidative model of retinal neurodegeneration induced by sodium iodate: morphofunctional assessment of the visual pathway. Antioxidants. (2023) 12:1594. doi: 10.3390/antiox12081594, PMID: 37627589 PMC10451746

[31] Enzbrenner A Zulliger R Biber J Pousa AMQ Schäfer N Stucki C . Sodium iodate-induced degeneration results in local complement changes and inflammatory processes in murine retina. Int J Mol Sci. (2021) 22:9218. doi: 10.3390/ijms22179218, PMID: 34502128 PMC8431125

[32] Yang Y Ng TK Ye C Yip YW Law K Chan S-O . Assessing sodium iodate–induced outer retinal changes in rats using confocal scanning laser ophthalmoscopy and optical coherence tomography. Invest Ophthalmol Visual Sci. (2014) 55:1696–705. doi: 10.1167/iovs.13-12477, PMID: 24526437

[33] Kolko M Vohra R van der Burght BW Poulsen K Nissen MH . Calcium-independent phospholipase A2, group VIA, is critical for RPE cell survival. Mol Vision. (2014) 20:511., PMID: 24791136 PMC4000714

[34] Schindelin J Arganda-Carreras I Frise E Kaynig V Longair M Pietzsch T . Fiji: an open-source platform for biological-image analysis. Nat Methods. (2012) 9:676–82. doi: 10.1038/nmeth.2019, PMID: 22743772 PMC3855844

[35] Militante J Lombardini JB . Age-related retinal degeneration in animal models of aging: possible involvement of taurine deficiency and oxidative stress. Neurochemical Research. (2004) 29:151–60., PMID: 14992274 10.1023/b:nere.0000010444.97959.1b

[36] Zhao J Kim HJ Sparrow JR . Multimodal fundus imaging of sodium iodate-treated mice informs RPE susceptibility and origins of increased fundus autofluorescence. Invest Ophthalmol Visual Sci. (2017) 58:2152–9. doi: 10.1167/iovs.17-21557, PMID: 28395299 PMC5389744

[37] Durlu YK Canbek S . Posterior segment findings in a patient with a CDHR1 biallelic pathogenic variant. Am J Ophthalmol Case Rep. (2024) 36:102228. doi: 10.1016/j.ajoc.2024.102228, PMID: 39737443 PMC11683250

[38] Foos TM Wu J-Y . The role of taurine in the central nervous system and the modulation of intracellular calcium homeostasis. Neurochem Res. (2002) 27:21–6. doi: 10.1023/A:1014890219513, PMID: 11926272

[39] Castelli V Paladini A d’Angelo M Allegretti M Mantelli F Brandolini L . Taurine and oxidative stress in retinal health and disease. CNS Neurosci Ther. (2021) 27:403–12. doi: 10.1111/cns.13610, PMID: 33621439 PMC7941169

[40] Marcinkiewicz J Grabowska A Bereta J Stelmaszynska T . Taurine chloramine, a product of activated neutrophils, inhibits *in vitro* the generation of nitric oxide and other macrophage inflammatory mediators. J Leucocyte Biol. (1995) 58:667–74. doi: 10.1002/jlb.58.6.667, PMID: 7499964

[41] Kim C Cha Y-N . Taurine chloramine produced from taurine under inflammation provides anti-inflammatory and cytoprotective effects. Amino Acids. (2014) 46:89–100. doi: 10.1007/s00726-013-1545-6, PMID: 23933994

[42] Jong CJ Azuma J Schaffer S . Mechanism underlying the antioxidant activity of taurine: prevention of mitochondrial oxidant production. Amino Acids. (2012) 42:2223–32. doi: 10.1007/s00726-011-0962-7, PMID: 21691752

[43] Gabrielian K Wang H-M Ogden TE Ryan SJ . *In vitro* stimulation of retinal pigment epithelium proliferation by taurine. Curr eye Res. (1992) 11:481–7. doi: 10.3109/02713689209001804, PMID: 1505195

[44] Bhavnani BR Cecutti A Gerulath A Woolever AC Berco M . Comparison of the antioxidant effects of equine estrogens, red wine components, vitamin E, and probucol on low-density lipoprotein oxidation in postmenopausal women. Menopause. (2018) 25:1214–23. 10.1097/GME.0000000000001222 30358716

[45] Feskanich D Cho E Schaumberg DA Colditz GA Hankinson SE . Menopausal and reproductive factors and risk of age-related macular degeneration. Archives of Ophthalmology. (2008) 126:519–24., PMID: 18413522 10.1001/archopht.126.4.519

[46] Schnabolk G Obert E Banda NK Rohrer B . Systemic inflammation by collagen-induced arthritis affects the progression of age-related macular degeneration differently in two mouse models of the disease. Invest Ophthalmol Visual Sci. (2020) 61:11–1. doi: 10.1167/iovs.61.14.11, PMID: 33289791 PMC7726584

[47] Yang X Rai U Chung J-Y Esumi N . Fine tuning of an oxidative stress model with sodium iodate revealed protective effect of NF-κB inhibition and sex-specific difference in susceptibility of the retinal pigment epithelium. Antioxidants. (2021) 11:103. doi: 10.3390/antiox11010103, PMID: 35052607 PMC8773095

[48] Machalińska A Lubiński W Kłos P Kawa M Baumert B Penkala K . Sodium iodate selectively injuries the posterior pole of the retina in a dose-dependent manner: morphological and electrophysiological study. Neurochem Res. (2010) 35:1819–27. doi: 10.1007/s11064-010-0248-6, PMID: 20725778 PMC2957578

[49] De Vera Mudry MC Kronenberg S S.-i. Komatsu and GD . Blinded by the light: retinal phototoxicity in the context of safety studies. Toxicol Pathol. (2013) 41:813–25. doi: 10.1177/0192623312469308, PMID: 23271306 PMC3786130

[50] De La Paz MA Zhang J Fridovich I . Antioxidant enzymes of the human retina: effect of age on enzyme activity of macula and periphery. Curr eye Res. (1996) 15:273–8. doi: 10.3109/02713689609007621, PMID: 8654107

[51] Anderson BD Lee TT Bell BA Wang T Dunaief JL . Optimizing the sodium iodate model: Effects of dose, gender, and age. Exp eye Res. (2024) 239:109772. doi: 10.1016/j.exer.2023.109772, PMID: 38158173 PMC10922497

